# Gentamicin delivery to the inner ear: Does endolymphatic hydrops matter?

**DOI:** 10.1371/journal.pone.0207467

**Published:** 2018-11-15

**Authors:** Pedro Marques, Maoli Duan, Nicolas Perez-Fernandez, Jorge Spratley

**Affiliations:** 1 Department of Otorhinolaryngology, S.João Hospital Centre, Porto, Portugal; 2 Unit of Otorhinolaryngology, Department of Surgery and Physiology, University of Porto Medical School, Porto, Portugal; 3 Department of Clinical Science, Intervention and Technology, Karolinska Institutet, Stockholm, Sweden; 4 Department of Otolaryngology, Head and Neck Surgery, Karolinska Universisty Hospital, Karolinska Institutet, Stockholm, Sweden; 5 Department of Otorhinolaryngology, Clinica Universidad de Navarra, Madrid, Spain; 6 Center for Health Technology and Services Research (CINTESIS), University of Porto Medical School, Porto, Portugal; Laurentian, CANADA

## Abstract

**Introduction:**

Middle ear application of gentamicin is a common medical treatment for uncontrolled Ménière’s disease. The objective of the study was to evaluate the impact of endolymphatic hydrops on inner ear delivery.

**Methods:**

Perilymph gentamicin concentrations and correlation with endolymphatic hydrops in an animal model were assessed. A group of 24 guinea pigs was submitted to surgical obstruction of the endolymphatic sac and duct of the right ear. Gentamicin was applied either to the right ear’s round window niche or through a transtympanic injection. Perilymph specimens were collected at different times. Histologic morphometry was used to evaluate both turn-specific and overall hydrops degree.

**Results:**

In animals with endolymphatic hydrops, lower concentrations of gentamicin were observed after 20 or 120 minutes of exposure and in both types of administration, when compared to controls. This difference reached statistical significance in the round window niche application group (Mann-Whitney, p = 0,007). A negative correlation between perilymphatic gentamicin concentration and hydrops degree could be observed in both groups, after 120 minutes of exposure (Spearman correlation, round window niche p<0,001; TT p = 0,005).

**Conclusions:**

The study indicates that the endolymphatic hydrops degree has a negative interference on the delivery of gentamicin into the inner ear following middle ear application.

## Introduction

Ménière’s disease (MD) is described by episodic vertigo associated with low/medium-frequency sensorioneural hearing loss and fluctuating symptoms (hearing, tinnitus and full and/or fullness) in the affected ear [1]. This disorder has been associated with an increased volume of endolymph in the membranous labyrinth–endolymphatic hydrops (EH). Post-mortem light microscopy assessment of the inner ear in patients with MD has shown that EH is the most frequent histopathologic finding: in all cases in the cochlea, 50%-60% in the vestibule but rarely in the canals [2]. As a consequence, EH has been considered for many years as its cause, despite not completely explaining every clinical feature of the disease [3]. More recent research has shown that EH is not mandatory, although found in every case of MD, when the 1995 guidelines are carefully applied, and sometimes also present in asymptomatic patients. EH must be necessary but not sufficient to cause MD, a point currently under debate [4].

As the precise ethiopathogenesis of MD has not been elucidated yet, an effective therapy has not been established, except for the control of symptoms via severity and rate of the vertigo episodes [5]. Variability through its course, from its basic pathophysiology to the clinic and treatment, indicates the natural course of the disease [6, 7] and a multiplicity of conservative approaches have been the mainstay of initial therapy [8]. Despite several published studies, an efficient evidence-based treatment is yet to be established.

Transtympanic (TT) administration of aminoglycosides and corticosteroids has proved to be an efficient method for intractable MD aiming to control vertigo episodes after a partial/total vestibular end-organ ablation [5], as with gentamicin [9–12]. A well-defined and consensual treatment regimen, as well as the method of administration, have not yet been designed. Different practices have been attempted to accomplish the best control of vertigo against the least damage to the hearing, but there is still no widely accepted standardized protocol [5]. The variability in individual responses may result from different factors, such as the susceptibility of the inner ear to drugs, the length of time in which a drug is in contact with the round/oval windows and the anatomic conditions involved [13].

In a healthy inner ear, the pharmacokinetics of different agents applied in the middle and inner ear have been intensively studied [13–20]. However, in inner ears with confirmed histopathological EH, the number of published studies is limited and, to our knowledge, the degree of EH on impacting the pharmacokinetics of drugs has not been studied [21, 22]. Thus, the aim of the present study was to understand how the EH degree influences the course of drugs, such as gentamicin, to the inner ear, after middle ear delivery.

## Material and methods

### 2. 1. Animals

A total of 44 Duncan-Hartley strain guinea pigs (Charles River Laboratories, France) with a positive Preyer’s reflex and weighing approximately 300g were used in this experiment. The Ethical Committee of the University of Porto Medical School approved the use and care of animals in accordance with the European Union directive 2010/63/EU for animal experiments (Project 8.2014). After admission, the animals were given a week to acclimate to the environment. Twenty-four guinea pigs underwent surgical obliteration of the endolymphatic duct of the right ear, as described below. After surgery, the animals were kept in the animal house for six weeks, allowing EH to develop. The remaining 20 animals were used as controls. The guinea pigs were accommodated two animals in each cage, in temperature (21–23°C) and humidity (45–65%) controlled conditions and light/dark cycle (12/12 h) controlled rooms. The animals had access to food and water ad libitum and ascorbic acid supplement was administered. Post-surgical surveillance of all animals was performed each 4 hours, on the first day, by a veterinarian. Subsequently, animals were routinely checked each 6 hours by one of the housing staff and twice a week by a veterinarian. An animal care table for animals submitted to surgical procedures, with items such as weight, physical appearance, clinical respiratory signs (respiratory rate), natural behavior and provoked behavior was filled on a daily basis.

### 2. 2. Surgical procedure for obliteration of the endolymphatic duct

For the induction of EH, surgical obliteration of the endolymphatic sac and duct of the right ear was performed via an extradural posterior fossa approach, as described by Kimura and Andrews [23, 24]. The left ear remained intact to be used as an internal control.

All animals were anesthetized after an intramuscular injection of ketamine (40 mg/kg) and peritoneal injection of xylazine (5mg/kg), for induction. Anesthesia was maintained with inhaled isoflurane 2,5%. Warming gloves were used to compensate hypothermia. Placed in a prone position with the neck slightly flexed, through a dorsal midline scalp incision, the guinea pig’s occiput was exposed. Through an extradural posterior cranial fossa approach, after exposure of the sigmoid sinus and moving it suitably medially, the bony operculum was identified. A 0.5mm burr (Nouvag, Goldach, Switzerland) was used to drill from the medial to the operculum and into the endolymphatic sac and duct. The endolymphatic duct was then packed with aseptic bone wax using a straight pick and an otologic elevator and the skull defect was reinforced with Gelfoam (Pfizer, New York), according to a technique described elsewhere, and the wound was closed [25]. Postoperative analgesia with tramadol (5-10mg/kg 2id) was administered in the first 48 hours. Animals were adequately hydrated during the postoperative period through subcutaneous injection of saline solution. All patients underwent postoperative oral antibiotics (enrofloxacin 5mg/kg bid). After a 48-hour period, the animals were re-joined with the others.

The surgical procedures were performed under sterile conditions and microscopic magnification using a *Carl Zeiss* Opmi Pico surgical microscope (Carl Zeiss, Oberkochen, Germany)

### 2.3. Experimental groups

To simulate the standard clinical procedure, gentamicin was transtympanically injected. For a more selective method, gentamicin was applied only to the RW niche.

#### 2.3.1. Group 1 –Round window niche gentamicin placement

In a left decubitus position, after a retroauricular approach, the tympanic bulla was exposed and opened. With an insulin syringe, a 25G lumbar puncture needle (BD Spinal Needle, Spain), 1 drop (around 60μl = 2,4mg) [26] of a solution of gentamicin sulphate (40 mg/ml; Gentamicina MG Labesfal, Labesfal Farma, Portugal) was placed in the right RW niche. The needle was angled 45° before administration and the syringe was systematically kept horizontally, which gave the final angulation of 45°. This angulation was considered anatomically appropriate to approach the round window niche. Caution was taken to avoid fluid leaking from the RW.

The animals were separated into two experimental groups (six in each) according to time to perilymph collection: 1A 20 minutes delay and 1B 120 minutes delay. During this period, the anesthetized animals were maintained in left decubitus position.

At the scheduled time, a single 2μl perilymph sample was collected from the RW through a 26G microlancet tip (BD Microlance, Spain) adapted to a P10 micropipette (VWR International, USA). The bulla was rinsed with saline solution and carefully dried before the sample was taken. Immediately after, the same procedure was performed in the left ear. The samples were stored at -80°C, in a cryotube containing 250μl of artificial perilymph solution.

Two non-operated control groups of three animals each were submitted to precisely the same procedures (groups 1ACTRL—20 minutes and 1B CTRL– 120 minutes)

In a distinct group of eight animals, where no EH was induced, perilymph was collected through a cochleostomy after 20 minutes (group 1C CTRL) or 120 minutes (group 1D CTRL) to assess possible contamination inaccuracies during the perilymphatic fluid sampling and to attest the quality of the technique employed. A superficial cochleostomy was performed at the basal turn of the cochlea, 2-3mm from the RW, with a 1mm diamond burr, and completed with a 26G microlancet tip (BD Microlance, Spain) adapted to a P10 micropipette (VWR International, USA), for a single 2μl perilymph sample collection.

#### 2.3.2. Group 2 –Transtympanic gentamicin injection

In a left decubitus position, with an ear speculum and an insulin syringe with a 25G (BD Spinal Needle, Spain) lumbar puncture needle, 0,12ml (4,8mg) of a solution of gentamicin sulphate (40 mg/ml Gentamicina MG Labesfal, Labesfal Farma, Portugal) was injected in the right middle ear.

Twelve animals were separated into two experimental groups according to time of perilymph collection– 2A (n = 6) (20 minutes) and 2B (n = 6) (120 minutes). The left ear was used as an internal control. In the respective schedule the tympanic bulla was opened through a retroauricular approach. Subsequently a 2μl perilymph sample was collected from the round window using the same technique as in group 1.

Two non-operated control groups of three animals each were submitted to the same procedures (groups 2A CTRL—20 minutes and 2B CTRL– 120 minutes).

### 2.4. Histological processing

After the experimental procedures, the animals were terminally anesthetized with sodium pentobarbital (Eutasol, Esteve Farma, Spain) intraperitoneally (33mg/kg). Under deep anaesthesia, the animals were transcardially perfused with normal saline complemented with heparin (10 units/L), followed by 1,000 millilitres of 4% paraformaldehyde in 0.1M phosphate buffer. The fixed animals were decapitated and both temporal bones dissected and post fixed in the same fixative for 48 hours. Following decalcification with Immunocal (Fisher Scientific, Portugal) for 48 hours, the temporal bones were embedded in soft Epon (Agar 100 resin kit, AgarScientific, UK). Ten-micrometre thick sections were cut with a tungsten carbide knife (C profile) along the midmodiolar plane. Every slide, in which all turns along the midmodiolar plane were observed, was mounted in glass slides and stained with 1% toluidine blue for light microscopy histologic and morphometric analysis.

### 2.5. Hydrops quantification

Sections were photographed with a *Leica* EC3 Camera (Leica microsystems, Switzerland) connected to a Zeiss Axioscope 40 microscope (Carl Zeiss, Germany) with the *Leica* Application suite Version 4.6.0 (Leica microsystems, Switzerland). All image analysis was prepared with *Image 1*.*50i* software (National Institutes of Health, USA).

#### 2.5.1. Hydropic ratio

Hydrops was determined by a previously tested method, which has been described in detail before [27, 28]. Each slide in which it was possible to measure, on both sides of the *modiolus*, the area of the *scala media* (SM) and the *scala vestibuli* (SV) was evaluated. To estimate a relative measure of the hydrops’ degree in the operated versus the control ear, the “proportion *scala media”* (PSM) was calculated as: PSM = SM area/ (SM area + SV area) [27, 28]. This comparative measurement compensates minor deviations in the plane of section between different ears [28]. The values from each turn, from all slides, were summed up to give the average PSM for each cochlear turn. A relative measure of hydrops was calculated between the right (operated) and the left ear (control), which was denominated—hydropic ratio (HR). An HR equal or inferior to 1 indicated that no hydrops had developed and an HR superior to 1 would signify an increase in SM’s volume in the operated ear in comparison to the control ear. As these proportions were calculated both in specific turns and overall, this method allowed not only an overall HR but also a turn-specific assessment.

### 2.6. Gentamicin dosage

An Architect iGentamicin assay (Abbott Laboratories), which is an *in vitro* chemiluminescent micro-particle immunoassay for the quantitative determination of gentamicin in human serum or plasma, was used. The measuring range of the iGentamicin assay was 0.3–10 μg/ml. The samples were adequately diluted with artificial perilymph (KCl (3.5mM), NaCl (125mM), NaHCO3 (25mM), CaCl2 (1.3mM), MgCl2 (1.2mM), NaH2PO4 (0.75mM), Dextrose (5mM)) [18] to keep this range, as appropriate. The final perilymphatic concentration was calculated taking into consideration these dilution rates.

### 2.7. Statistical analysis

Categorical variables were described in absolute (N) and relative frequencies (%), whereas continuous variables were described in average plus standard deviation (SD) or median and percentile. Continuous variables without a normal distribution were analysed with non-parametric tests of Mann-Whitney. To evaluate the strength of an association between two continuous variables a correlation of Spearman was applied to compensate for the biased nature of the variables involved. In every test, it was considered a confidence level of 95% (significance p<0,05). The analysis was performed with SPSS v.24.0 (Statistical Package for the Social Sciences, Chicago, IL, USA).

## Results

### 3.1. Evaluation of induced endolymphatic hydrops

**Fig 1:**
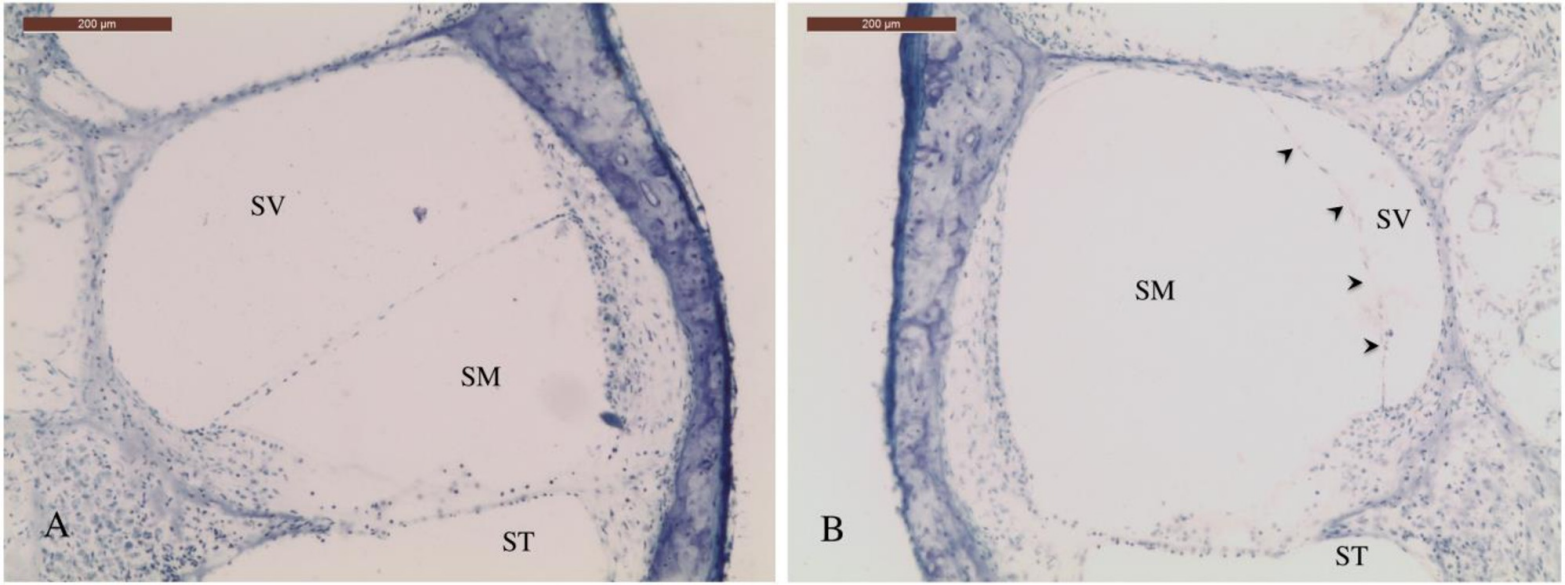
Midmodiolar sections of the cochlea. Second turn of the left (A) and right (B) cochlea from animal #13, under light microscopy and toluidine blue stain. Arrowheads point the dilation of the Reissner’s membrane (B). (SM: *scala media*, SV: *scala vestibuli*, ST: *scala tympani*).

A successful surgical obstruction of the right endolymphatic sac and duct, histopathologically expressed by EH on light microscopy, was confirmed in all experimental animals (Fig 1B).

A slight to severe hydrops occurred in all dissected ears, with a HR average of 1,39 (Wilcoxon test, p<0,001) (Table 1). No EH was observed in any of the control cochleae. One cochlea (animal #17) was not appropriate for histologic analysis due to inadequate fixation.

**Table 1:** Endolymphatic Hydropic Ratio between the right and left ear.

Cochlea	Min*	Average*	Max*	Wilcoxon test (p value)
**Turn 1 (N = 19)**	1,04	1,37	1,92	p<0,001
**Turn 2 (N = 23)**	1,04	1,40	2,32	p<0,001
**Turn 3 (N = 23)**	0,93	1,44	2,28	p<0,001
**Overall (all turns)**	1,11	1,39	2,13	p<0,001

*by measurement of PSM *(scala media* area/(*scala media* + *scala vestibuli*); turns numbered from the base of the cochlea; N, number of animals.

### 3.2. Perilymphatic gentamicin concentration by method and time period of administration

Overall, following the delivery of gentamicin to the inner ear, lower levels of the drug were found in ears with histologically confirmed EH in comparison to the controls, which did not undergo endolymphatic sac and duct surgery (Table 2).

**Table 2:** Perilymphatic gentamicin concentration in the studied groups.

				HE					CTRL			
Administration Technique	Time (Minutes)	N	Right ear Average (μg/ml)	SD (μg/ml)	Left ear Average (μg/ml)	SD (μg/ml)	N	Right Ear Average (μg/ml)	SD (μg/ml)	Left ear Average (μg/ml	SD (μg/ml)	Mann-Whitney test (p-value)
**Round window niche**	**1A** **20 min**	5*	772,75	429,98	0,01	0,14	3	2030,42	521,79	0,01	0,01	**0,007**
	**1B** **120 min**	6	2757,11	282,79	0,00	0,00	3	3061,33	521,79	0,00	0,00	**0,007**
	**1C** **20 min** **Cochleostomy**	-	-	-	-	-	4	1991,53	284,41	0,00	0,00	p> 0,05**
	**1D** **120 min** **Cochleostomy**	-	-	-	-	-	4	3050,00	61,68	0,00	0,00	p> 0,05**
**Transtympanic**	**2A** **20 min**	6	1888,11	263,99	0,00	0,00	3	2970,31	933,68	0,02	0,045	0,197
	**2B** **120 min**	6	3273,87	230,99	0,005	0,005	3	3542,24	76,03	0,01	0,01	0,071

* 1 animal excluded due to due to inadequate fixation

** comparisons between groups 1C/1A and 1B/1D

CTRL, control; EH, endolymphatic hydrops; min, minutes; N, number of animals; SD, standard deviation

**Fig 2:**
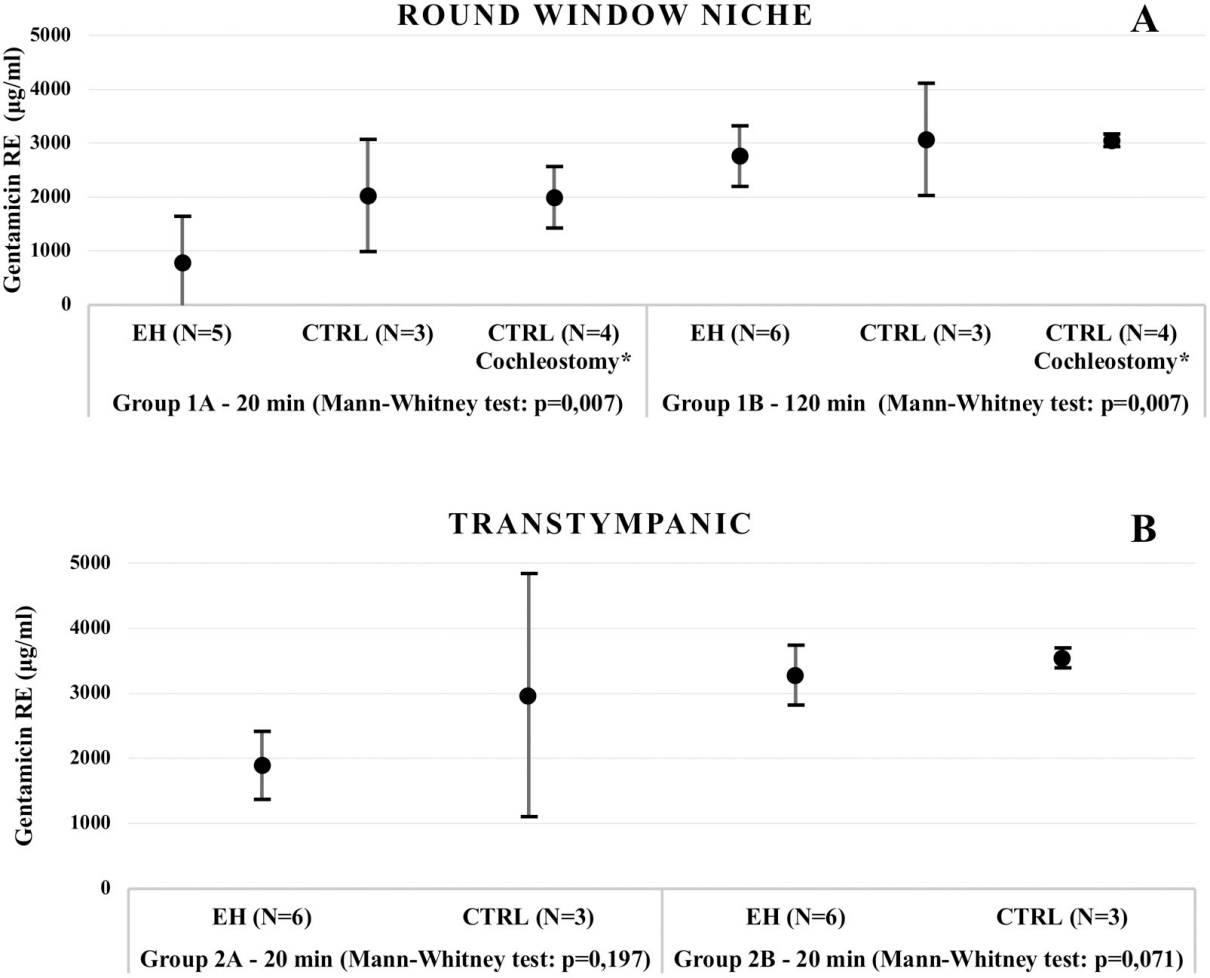
**Perilymphatic gentamicin concentration after RW niche application (A) and Transtympanic application (B).** Data presented as average ± 2 standard deviation. (RE–right ear; CTRL–control; EH–endolymphatic hydrops; min- minutes; N–number of animals; 20 min–time period of administration of 20 minutes; 120 min–time period of administration of 120 minutes).

This difference was statistically significant when gentamicin was specifically delivered to the RW niche, in both time periods of 20 and 120 minutes (Mann-Whitney, p = 0,007), whereas a trend was observed in the TT injection groups. (Fig 2A and 2B).

None of the left ear perilymph samples elicited significant levels of gentamicin, as only vestigial concentrations were detected.

### 3.3. Correlation between hydropic ratio and perilymphatic levels of gentamicin

**Fig 3:**
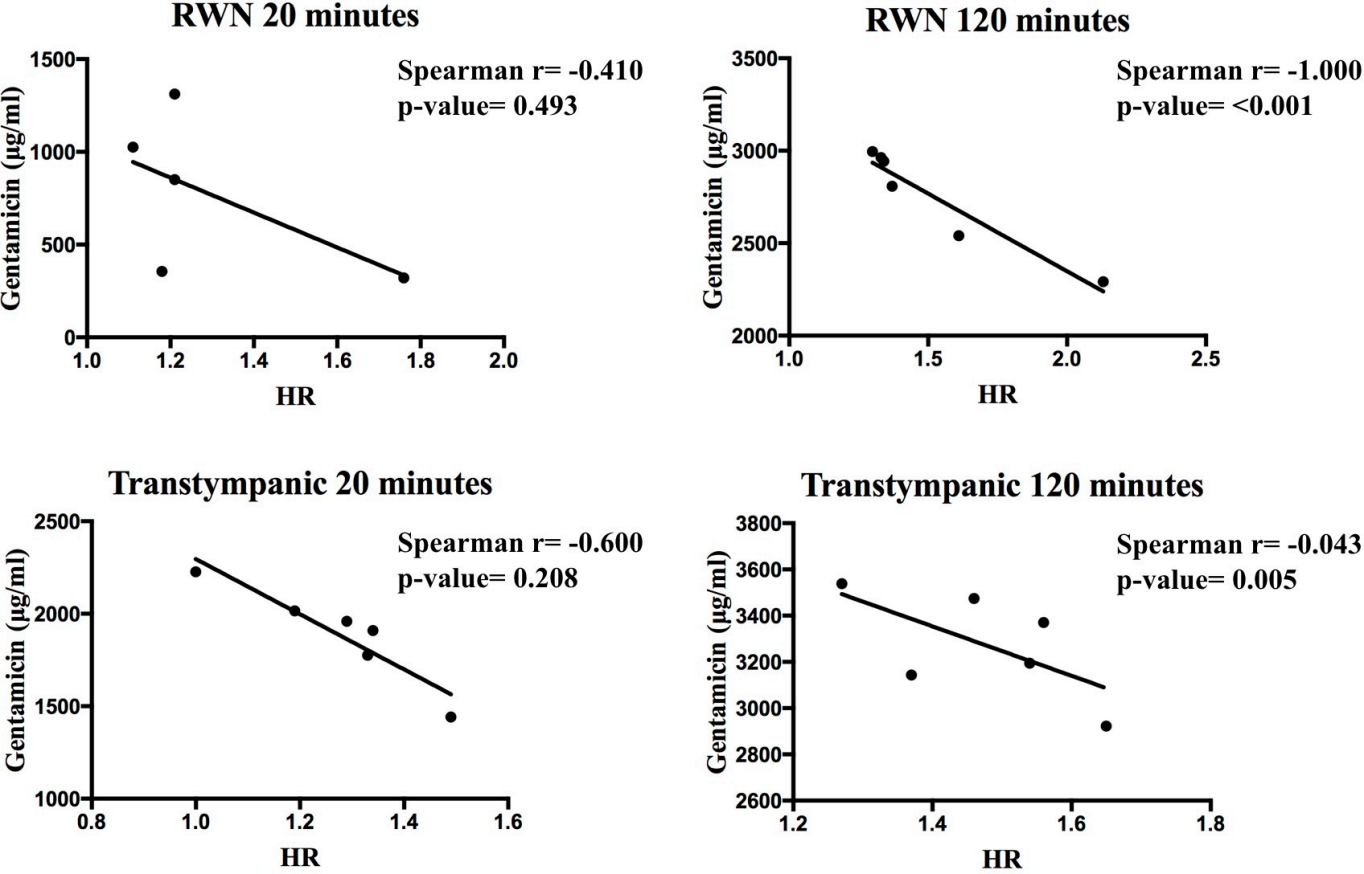
Correlation coefficients between Hydrops Ratio and perilymphatic gentamicin concentration. Correlation coefficients (Spearman correlation) in different times periods and methods of administration. (RWN–round window niche; HR–hydropic ratio).

When the gentamicin concentrations in perilymph were matched with the HR a negative correlation was observed in both groups in which perilymph samples were collected after 120 minutes of gentamicin exposure (RW niche: p<0,001; TT: p = 0,005) (Fig 3).

## Discussion

This work showed that EH has a negative impact on the gentamicin delivery to the inner ear when compared with control animals. This assumption was stronger when the administration was limited to the round window niche but also perceptible after TT injection. The observed negative correlation values when perilymph collections were performed after 120 minutes of exposure (RW niche p<0,001, TT p = 0.005) appear to support this statement.

In the animal model of EH, the perilymphatic concentration of drugs administered to the middle ear depends on factors such as the time course in the middle ear, entry through the RW and oval window membranes, dilution effects of cerebrospinal fluid (CFS) and elimination to blood or tissues [18, 20]. Factors such as size and thickness of the membrane, electrical charge and substance solubility are assumed to also affect permeability [29–31].

It has been previously recognized that intratympanically administered drugs reach the inner ear compartment mainly through two passageways: the round window (57%) and the oval window ligament (35%) [18]. Intrinsic properties of the RW membrane, namely an increase in its thickness by a hyperplastic middle fibrous layer, as seen in EH [32], and other inflammatory conditions of the middle ear [33–35] may influence this permeability. Also, the properties of the outer epithelium of the RW membrane allow a dynamic passage of substances, rather than just a simple connective tissue boundary, and have been considered markedly significant and sensible to manipulations [20]. A still open question is if these permeability mechanisms occur in a similar degree in the structures of the oval window region and which role they represent in intratympanic drug delivery into the inner ear.

The longitudinal distribution along the cochlea, as well as a communication between cochlear *scalae*, could also have an important impact on the pharmacokinetics of drugs. Evidence on local interscala communications has been provided [36], as well as conceivable connections between the perilymphatic and endolymphatic spaces, at least after systemically administered drugs [18, 37, 38], though the epithelial cells lining the endolymph compartment have been described to fairly limit solute movements [20].

As such, drug levels on the perilymph may depend not only on how fast it passes from the middle to the inner ear but also on how quickly it communicates with these different spaces or is cleared. Significantly, substances entering the cochlea from the middle ear appear to be mostly confined to the basal turn, rather than reaching the upper turns of the cochlea [39]. Therefore, the existence of this basal-apical gradient along the cochlea appears to be critical in explaining how vestibular function can be ablated in humans while preserving the function of speech frequency regions of the cochlea [18, 40, 41]. However, this particular detail has not yet been addressed in EH.

An increased area of a hydropic membranous labyrinth may potentially increase the gentamicin way to the endolymph, reducing its perilymphatic concentration. In the cochlea, Reissner’s membrane is a dynamic structure involved in ion-fluid transportation [42] and changes in cellular morphology in the hydropic ear have been described. Absence of mesothelial cells and enlarged epithelial cells of the endolymphatic surface [43], suggesting a “stretched” rather than “grown” membrane [44], as well as intercellular gaps have additionally been described [43]. However, no significant differences between the tight junctions of epithelial cells were found [45]. Despite the ongoing controversy, it is conceivable that a reduced passage of gentamicin to the perilymph in a hydropic ear, based on pressure issues, could also play a role, at least when Reissner’s membrane loses its high compliance. This might occur following long-standing distension and volume increase, leading to important endolymphatic pressure gradients [46].

Conceivably, all or at least some of these modifications in Reissner’s membrane may influence fluid changes between perilymphatic and endolymphatic spaces [47] and have not yet been accounted for the impact on drugs’ distribution in the inner ear.

Electrophysiological changes may also play a role in the pathophysiology of EH, namely the endolymphatic potential decrement [48]. This would support the theory that, in EH, an increased flow of drugs from the SV to the endolymph, against a reduced endocochlear potential could occur, adding to a further reduction of the concentrations of drugs available in the SV and, due to the multiple communicating spaces, in the whole perilymphatic space [18].

Still, the understanding of pharmacokinetics of drugs in the inner ear is probably not restricted to open fluid spaces of endolymph and perilymph. The soft tissues of the membranous labyrinth to which drugs can distribute, should also play a role, with interference in perilymphatic concentrations. In this line, a recent guinea pig study, estimated that this could represent up to 24% of the total inner ear volume [20]. Nevertheless, in the specific model of EH, this role has not been studied, yet.

Previous research in human series have likewise addressed drug delivery to the inner ear. In this context, studies of magnetic resonance imaging (MRI) with gadolinium (Gd) have revealed differences in the entrance of Gd to the inner ear that correlated with the severity of EH, although most were associated with the vestibule region. Shi and colleagues observed a compromised distribution of Gd across the oval window, which also correlated with the severity of EH [21]. Further MRI studies, either in animals or humans, showed that Gd signal was higher in the vestibule than in ST of the cochlea [21, 49, 50]. To better understand this finding, it has been suggested that the perilymphatic space in the vestibule of patients with severe EH could be exceptionally compressed and could therefore explain the compromised route for Gd diffusion [21]. However, this reduced passage to the inner ear appears not to be limited to the oval window region, with Yoshioka et al. having reported that the RW’s permeability and the passage to the ST after TT injection of Gd was also compromised in patients with MD [51].

When a hydropic membranous labyrinth is discussed, as in MD, anatomic distortions are implicit, and appear to occur in a predictable manner, with a pattern suggesting a progression from the cochlea and saccule [2]. A greater hydrops degree may position membranes closer to the round and oval window regions. The question arises as to whether this anatomical feature could facilitate the absorption of drugs to the endolymph. If in closer contact with both window membranes, there could occur an almost directly passage from the middle ear to the endolymph. These changes would help explaining the reduced concentration of gentamicin observed in the perilymphatic space and its increase in the endolymph.

According to recent evidence, a blood-labyrinth barrier dysfunction, in the microvasculature of vestibular end organs, has also to be accounted for. As such, MRI studies in MD patients revealed a blood labyrinth breakdown with an associated increase in contrast permeability. This was observed not only in the symptomatic but also in the asymptomatic ear, suggesting a systemic abnormality which could potentially be considered, in the future, a biomarker of the disease [52]. This observation likely contributes to edematous changes in the underlying stroma, vacuolization and an increase in vesicles, with transcytosis of macromolecules [53]. Thus, this increased permeability could play a significant part in the drug loss from the labyrinth spaces.

Altogether, this results and previous observations, both in animals and in humans, complementarily demonstrate that a reduction of the flow from the middle to the inner ear could exist and correlates with the EH state.

In spite of an apparently reduced absorption in EH, as supported by this study, Kimura et al., after lateral canal application of gentamicin, have observed higher sensitivity in hydropic ears. He observed an increase of lesions in all sensory epithelia and in particular in the organ of Corti, noting the apparent hypersensitivity of the hydropic inner ear to external aggressions, including sound exposure, aminoglycosides, certain diuretics and hypoxia [22]. Although the precise mechanism of increased ototoxicity on the hydropic ears is not clear, Kimura argued that ototoxic drugs could enter more readily into the endolymphatic space and remain longer in the endolymphatic compartments due to a possible decrease in absorption of endolymph in the hydropic condition [22].

As noted above, there are still many unanswered questions on this topic. This concept of communication between inner ear spaces and the possible impact of EH has the potential to be one of the research questions in the near future and should be explored. To our knowledge, this is the first published report on gentamicin delivery to the inner ear in an EH guinea pig model.

Possible limitations of the current study may include the research model used, with the inherent difficulties of the surgical procedure and the long period of follow-up, in which variable degrees of hydrops were observed in different animals. If a greater series of animals had been used the power of the study might have been higher, but this possibility was excluded for ethical and economic reasons.

An additional concern was the quantification of the dose of gentamicin administered. Based on the study of Tripp *et al*. [26] it was assumed that a volume of 1 drop was approximately 60μl, corresponding to 2400μg of Gentamicin. The needle orientation, at the time of drop release, was considered to be an important factor in determining the drop volume. For that purpose, a standardized 45° was carefully kept in every experiment but, by the results achieved, it should considered that the volume provided could be, in fact, slightly higher than that presented by Tripp *et al*. [26]. Still, as every experiment was performed equally, it was considered that it had no impact on the conclusions retrieved.

Finally, perilymph collection and analysis are delicate procedures, demanding a high degree of precision. Possibly, the use of the microdialysis technique could have contributed to an increased accuracy of the collection [54], however, under the current study paradigm, our focus was in concentrations following perilymph harvest at a single time. The procedure and place of collection might have involved a minor risk of contamination of cerebral-spinal fluid as, after a 2μL collection near the round window, we could estimate that only 60% would correspond to perilymph [55]. The results showed gentamicin concentrations slightly above what would be expected, though similar results were presented by Hibi *et al*. [13]. Experimentally, a minor perilymph leaking was only observed after removing the microlancet tip from the RW membrane and not before it, Though, it can reasonably be assumed that, with the technique applied, the CSF wash could have been realistically reduced, and the purity of the sample could actually be significantly superior to 60%. Moreover, if accounted for its actual absolute impact, it would certainly lead to a decreased concentration of gentamicin, proportional to the volume collected [55] but, affecting in a similar way, both cases and controls. As this study was essentially focused to the relative values between EH and controls, it is believed that did not prejudice the goal of the current research.

## Conclusions

Middle ear application of gentamicin is a common medical treatment in uncontrolled MD. Current protocols are not consensual and have been developed on the basis of studies that did not consider the EH degree. This study demonstrates that EH degree has a clear negative correlation with the delivery of gentamicin to the perilymph, when compared to a normal ear.
